# Large renal angiomyolipomas in tuberous sclerosis

**DOI:** 10.1093/omcr/omad019

**Published:** 2023-03-25

**Authors:** G Esson, P Haslam, J A Sayer

**Affiliations:** Renal Services, The Newcastle upon Tyne Hospitals NHS Foundation Trust, Newcastle upon Tyne, UK; Department of Radiology, The Newcastle upon Tyne Hospitals NHS Foundation Trust, Newcastle upon Tyne, UK; Renal Services, The Newcastle upon Tyne Hospitals NHS Foundation Trust, Newcastle upon Tyne, UK; Translational and Clinical Research Institute, Newcastle University, Newcastle upon Tyne, UK; National Institute for Health and Care Research, (NIHR) Newcastle Biomedical Research Centre, Newcastle upon Tyne, UK

**Figure 1:**
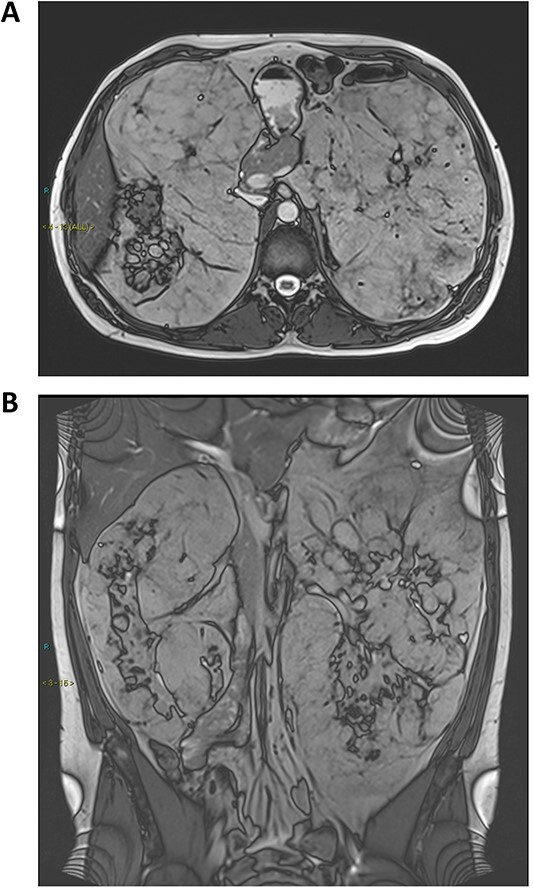
(**A**) Axial and (**B**) coronal sections from an abdominal MRI scan showing bilateral massive renal angiomyolipomas and loss of normal kidney anatomy.

A 32-year-old man with a known diagnosis of tuberous sclerosis presented with early satiety and abdominal discomfort. Investigations revealed multiple large angiomyolipoma arising from and surrounding both kidneys. Axial and coronal true fast imaging with steady state precession magnetic resonance imaging (MRI) scans (Fig. 1) showed that the renal parenchyma was compressed and dispersed by multiple, large, inseparable, predominantly fatty angiomyolipomas. This is not the typical appearance of well-demarcated renal angiomyolipomas associated with tuberous sclerosis. The normal renal anatomy and corticomedullary differentiation were not discernable and the abdominal contents were displaced. Kidney function was preserved with an estimated glomerular filtration rate (eGFR) greater than 90 ml/min/1.73 m^2^ and urine dipstick was negative. Molecular genetic testing confirmed a *de novo* heterozygous pathogenic variant in *TCS2*. The patient was commenced on everolimus and has had no complications.

Angiomyolipomas are a common feature of tuberous sclerosis, occurring in 60–80% of patients [1] and are the leading cause of mortality due to rupture, bleeding and kidney failure [2]. Renal angiomyolipomas greater than 4 cm in diameter are often symptomatic with haematuria, flank pain and a palpable mass [3]. Kidney cysts are seen in 30–45% of patients with tuberous sclerosis and are associated with kidney failure and hypertension [4]. Renal cell carcinoma is a rare complication of tuberous sclerosis, occurring in 2–3% of patients [4, 5]. Treatment with the mammalian Target of Rapamycin (mTOR) inhibitor everolimus is now first-line treatment for asymptomatic tuberous sclerosis-associated angiomyolipomas with a diameter of greater than 3 cm, and selective embolization and partial nephrectomy are second-line options [6]. Patients with a diagnosis of tuberous sclerosis should have annual assessment of kidney function (eGFR) and blood pressure and a MRI of the abdomen to assess kidney complications every 1–3 years [6].
